# Neonatal repetitive pain in rats leads to impaired spatial learning and dysregulated hypothalamic-pituitary-adrenal axis function in later life

**DOI:** 10.1038/srep39159

**Published:** 2016-12-14

**Authors:** Mengying Chen, Dongqing Xia, Cuiting Min, Xiaoke Zhao, Yinhua Chen, Li Liu, Xiaonan Li

**Affiliations:** 1 Department of Child Health Care, Children’s Hospital of Nanjing Medical University, Nanjing, China; 2 Department of Rehabilitation, Children’s Hospital of Nanjing Medical University, Nanjing, China; 3 Department of Applied Physics and Electronics, Umeå University, Umeå, Sweden

## Abstract

Preterm birth is a major health issue. As part of their life-saving care, most preterm infants require hospitalization and are inevitably exposed to repetitive skin-breaking procedures. The long-term effects of neonatal repetitive pain on cognitive and emotional behaviors involving hypothalamic-pituitary-adrenal (HPA) axis function in young and adult rats are unknown. From P8 to P85, mechanical hypersensitivity of the bilateral hindpaws was observed in the Needle group (*P* < 0.001). Compared with the Tactile group, the Needle group took longer to find the platform on P30 than on P29 (*P* = 0.03), with a decreased number of original platform site crossings during the probe trial of the Morris water maze test (*P* = 0.026). Moreover, the Needle group spent more time and took longer distances in the central area than the Tactile group in the Open-field test, both in prepubertal and adult rats (*P* < 0.05). The HPA axis function in the Needle group differed from the Tactile group (*P* < 0.05), with decreased stress responsiveness in prepuberty and puberty (*P* < 0.05) and increased stress responsiveness in adulthood (*P* < 0.05). This study indicates that repetitive pain that occurs during a critical period may cause severe consequences, with behavioral and neuroendocrine disturbances developing through prepuberty to adult life.

Preterm birth is a major health issue^1^. As part of their life-saving care, most preterm infants are required to spend several weeks in the neonatal intensive care unit (NICU) and are inevitably exposed to repetitive skin-breaking procedures^2^,^3^. Despite an increasing awareness regarding the consequences of non-intervened pain, the management of pain in newborns still depends on the hospital’s protocols and is not always adequate^2^,^3^,^4^.

The current view of pain is that it arises from a distributed network of brain activity, none of which is unique to pain, but when coordinated or synchronized, results in the sensory, emotional, motivational, and cognitive experience that is pain^5^,^6^. Therefore, non-intervened pain, particularly that which occurs during a critical period of brain development, can cause long-term behavioral alteration in humans and animals^7^,^8^,^9^. Most studies have focused on the alternation of pain responsiveness (hypoalgesia or hyperalgesia) caused by the plasticity in developing pain pathways^8^,^10^,^11^,^12^,^13^. Few reports have discussed the consequences of pain-related behavior^14^,^15^. Notably, such long-term alteration of pain-related behavior, including cognitive and emotional aspects, could have a great impact on neuropsychological development.

Clinical studies have noted that frequent skin-breaking procedures were associated with poor cognitive outcome and could predict higher internalizing behaviors at 18 corrected chronological age (CCA) months; these effects lasted to school age in children born very prematurely^15^,^16^,^17^. The alteration of executive function and internalizing behaviors might be associated with high risks of learning difficulty and deficits in social-emotional behaviors in later life^18^. Animal models are optimal for investigating the long-term effects in adulthood, but the findings are diverse. Anand’s group revealed that adult rats that experienced neonatal repetitive pain showed an increased preference for alcohol, increased anxiety and withdrawal behavior, and a lower threshold for learned helplessness^14^. By contrast, Murphy’s group discovered that neonatally injured adult rats had significantly decreased anxiety-like behaviors and decreased sensitivity to stress^19^. In addition, the impacts of neonatal pain on learning and memory development in adulthood are paradoxical^20^,^21^. Differences in the paradigms, the type of animals used, and exposure time to pain, sex, and the “critical window” when the pain occurred likely explain the heterogeneity of findings. The critical windows are the preterm period in human neonates and the first postnatal week in newborn rat pups. During this period, the brain is extraordinarily plastic and the nervous, endocrine and immune systems are all undergoing functional and structural development^22^; thus, dynamic observation of these changes could provide new insight into brain development under adverse conditions. A better understanding of developmental neuroendocrine functioning can be gained by investigating the cognitive and emotional behavior following postnatal early repetitive painful stimulation in young and adult rats.

The complexity of pain pathways suggests that pain processing is far beyond merely auditing pain intensity and location. The activation of the prefrontal cortex, the cingulate gyrus and insula cortex raises the distinct likelihood that higher order cognitive and emotional elements are involved with the processing of nociceptive stimuli^5^,^23^. The hippocampus, an important component of the limbic system, is not only involved in the pain processing but also plays an important role in the regulation of hypothalamic-pituitary-adrenal (HPA) axis activity^23^,^24^. Long-term neuroendocrine changes observed in developmental abnormalities associated with early traumatic events, such as child abuse and stress, might be attributed to the plasticity of the HPA axis^25^,^26^. Grunau *et al*. found that there was a shift in HPA responsiveness over time following high levels of exposure to neonatal pain, as the cortisol response was enhanced at 8 and 18 CCA months, but dampened at the neonatal period, 3 CCA months and school age^27^,^28^,^29^. Considering the HPA axis ontogeny, rat pups showed a markedly reduced adrenocortical response to stress during postnatal day 2 (P2) to P14; this period of adrenocortical quiescence has been termed the “stress hyporesponsive period” (SHRP)^30^. SHRP can be considered a protective mechanism, because rats exposed to high levels of glucocorticoids during the first week of life showed altered social behavior and learning performance^31^,^32^. Based on these discoveries, we hypothesize that rats subjected to repetitive needlestick protocol during the SHRP could exhibit increased vulnerability to long-term cognitive and emotional impairment.

Working in tandem with the mineralocorticoid receptor (MR), the glucocorticoid receptor (GR) is a crucial component of the HPA axis, controlling glucocorticoid feedback^33^,^34^. Notably, the GR is more ubiquitously distributed in the brain than the MR, and it is enriched in the hippocampus, the lateral septum and the paraventricular nucleus of the hypothalamus^35^. As a part of the stress response system, GR not only alters the level of glucocorticoids but also modulates cognitive functions and the processing of emotional stimuli^36^. Murphy’s group suggested that neonatal injury accelerated corticosterone negative feedback and resulted in compensatory changes of GR expression in adult rats^37^. However, it is unknown whether the developmental GR expression in the hippocampus is changed with behavioral performance in rats that experienced neonatal repetitive pain.

Given that the physical and behavioral maturity of rat pups during the first postnatal week is developmentally equivalent to that of preterm infants^38^, in the present study, rat pups subjected to a repetitive needlestick protocol during the first postnatal week were used to model preterm neonates in the NICU. Thus, the first purpose of this study was to examine the cognitive and emotional behaviors in both young and adult rats that experienced neonatal repetitive pain. The second purpose was to examine the HPA axis function in the stress response after neonatal repetitive pain from prepuberty to adulthood.

## Results

### Impacts of neonatal repeated pain on body weight gain

To explore the effect of neonatal repeated pain on physical development, rats were weighed every week following birth. The body weight gain between the two groups was similar (Table 1: F(age) = 2041.154, *P* < 0.001; F(age * group) = 0.692, *P* = 0.591), without a significant group difference (F(group) = 0.923, *P* = 0.346). However, the Needle group exhibited a temporary reduction in body weight gain on P8 (t = −6.672, *P* < 0.001) and P15 (t = −3.901, *P* < 0.001) compared with the Tactile group.

### Impacts of neonatal repeated pain on behavioral development

#### von Frey mechanical tests

The mechanical withdrawal threshold (MWT) to von Frey hair stimulation of the left hindpaw (F(age) = 34.835, *P* < 0.001; F(age * group) = 7.318, *P* < 0.001) and right hindpaw (F(age) = 39.458, *P* < 0.001; F(age * group) = 39.458, *P* = 0.001) increased in a similar manner over time (Table 2). However, in the Needle group, the MWT of the bilateral hindpaws was significantly lower than those in the Tactile group at every time point (left hindpaw: F(group) = 37.969, *P* < 0.001; right hindpaw: F(group) = 27.321, *P* < 0.001; both *post-hoc* test: *P* < 0.05).

#### Morris water maze (MWM) tests

**Figure 1:**
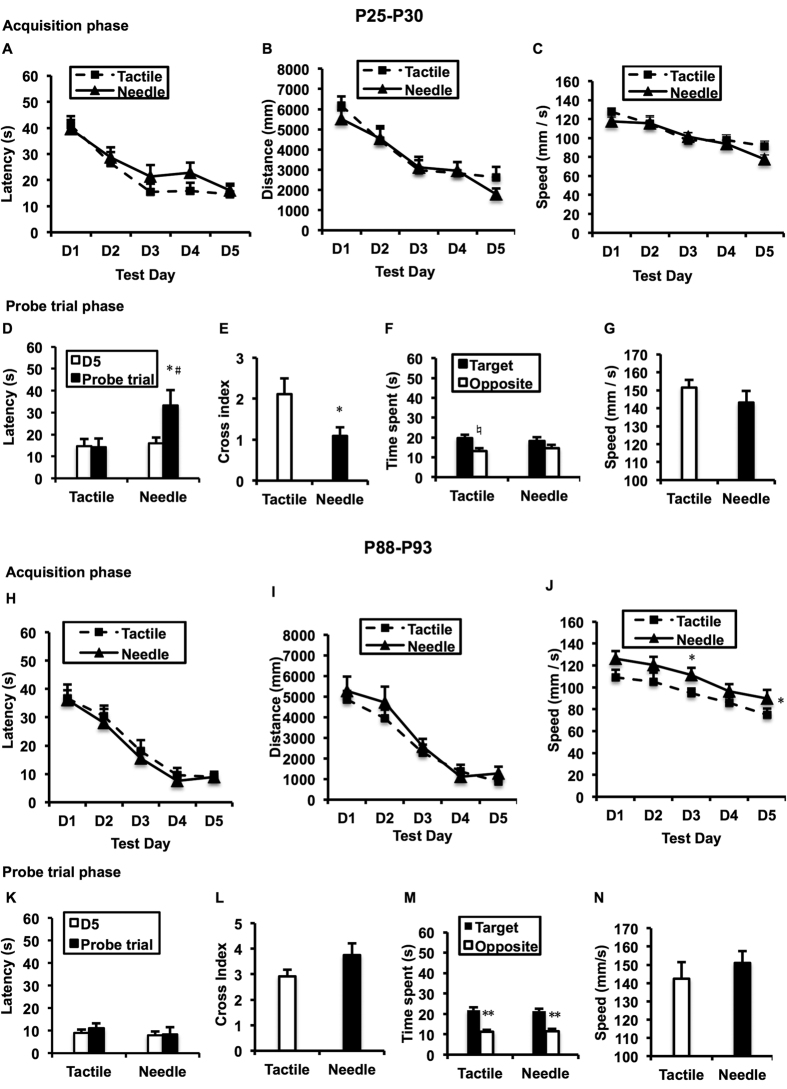
Morris water maze (MWM) performance. Repeated-measures ANOVA was used to determine the significance of differences in latency to find the hidden platform (**A**,**H**), distance (**B**,**I**), and speed (**C**,**J**) between the two groups on P25-29 and P88-92 during the acquisition phase. Two-way ANOVA was performed to compare the main effects of neonatal treatment and test day (**D**,**K**), as well as the main effects of neonatal treatment and quadrant on P30 and P93 (**F**,**M**). The paired t-test was used to assess the latency difference between two test days and the time difference between two quadrants. The independent t-test was used to detect group differences in crossing index (**E**,**L**) and speed (**G**,**N**) on P30 and P93 during the probe trial phase. Data are expressed as the mean ± SEM. *P < 0.05, **P < 0.01 for Needle vs. Tactile (n = 12). ^#^*P* < 0.05 for the last day of the acquisition phase vs. probe trial day (n = 12).

During the acquisition trials, the spatial-memory training of the MWM was effective (P25-P29: F(day) = 24.569, *P* < 0.001; P88-P92: F(day) = 38.738, *P* < 0.001), but failed to yield any significant differences in the time required to discover the escape platform between the Needle and Tactile groups on either P25-P29 (Fig. 1A: F(group) = 0.731, *P* = 0.403) or P88-P92 (Fig. 1H: F(group) = 0.839, *P* = 0.37). Similarly, no significant differences were found in the distance between the two groups during the two time points (*P* > 0.05). There was no difference in the swim speed between the groups during P25-P29 (*P* > 0.05). However, significant differences in the swim speed between the groups were found on P88-P92 (Fig. 1J: F = 4.687, *P* = 0.043), and the Needle group swam faster than the Tactile group, particularly on D3 (t = 2.098, *P* = 0.048).

During the probe trial phase, a significant group difference was detected in the time to find the original platform location between P29 and P30 (Fig. 1D: F = 4.866, *P* = 0.034). Although the Tactile group maintained a high level of performance between the training day (P29) and the probe trial day (P30) (t = −0.004, *P* = 0.997), the Needle group took longer to find the platform on P30 than on P29 (t = 2.526, *P* = 0.03). Additionally, the number of original platform site crossings in the Needle group was significantly lower compared with that of the Tactile group on P30 (Fig. 1E: t = −2.423, *P* = 0.026). As illustrated in Fig. 1F, the Tactile group spent more time in the target quadrant than in the opposite quadrant (t = 2.438, *P* = 0.041), whereas the Needle group spent similar amounts of time in the two quadrants on P30 (t = 1.158, *P* = 0.274); no differences in search pattern (F = 0.002, *P* = 0.967) and swim speed (Fig. 1G: t = 1.579, *P* = 0.129) between the two groups were found on P30.

However, on P93, both groups spent more time in the target quadrant than in the opposite quadrant (Fig. 1M: F = 68.256, *P* < 0.001; Tactile: t = 5.547, *P* < 0.001, Needle: t = 4.66, *P* = 0.001), and there was no difference in the quadrant search pattern between the groups (F = 0.018, *P* = 0.895). There were no significant differences in the parameters (*P* > 0.05 in all comparisons).

#### Open-field (OF) test

As shown in Table 3, the OF test showed that the proportion of distance spent in the central area (t = 2.231, *P* = 0.032) and the proportion of center entries (t = 2.863, *P* = 0.007) for the Needle group were significantly higher than those in the Tactile group, but there were no significant differences between groups in the total distance (t = 1.453, *P* = 0.154) and total entries (t = 1.334, *P* = 0.190) on P24. Moreover, there was a tendency toward more time spent in the central area for the Needle group than the Tactile group (t = 2.025, *P* = 0.05). In addition, there were no significant differences on other parameters (*P* > 0.05).

Similar to P24, the Needle group spent more time (t = 2.189, *P* = 0.036) and proportion of distance (t = 2.798, *P* = 0.009) in the central area and travelled for less time in the peripheral area (t = −2.506, *P* = 0.017) compared with the Tactile group on P87. In addition, the total entries for the Needle were significantly more than for the Tactile group (t = 2.720, *P* = 0.010). However, there were no significant differences on other parameters (*P* > 0.05).

### Impacts of neonatal repeated pain on HPA axis function

#### Changes in relative adrenal weight (mg/g)

**Figure 2:**
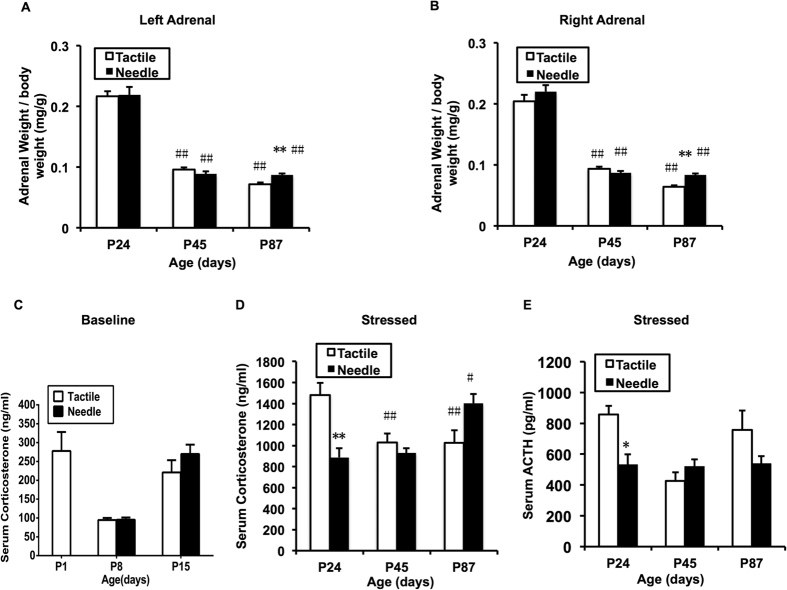
Relative weight of the adrenal glands, serum corticosterone and ACTH levels. The two-way ANOVA test was used to detect group differences in left (**A**) and right (**B**) adrenal weights to body weight, basal corticosterone level (**C**) and stress-triggered corticosterone level following 30 min of exploration in the open-field (**D**), and stress-triggered ACTH level (**E**) at different ages between the two groups. The LSD *post-hoc* test was used to detect group differences at each age. Data are expressed as the means ± SEM. *P < 0.05, **P < 0.01 for Needle vs. Tactile; ^#^*P* < 0.05, ^##^*P* < 0.01 compare to P24 (n = 10).

Bilateral adrenal glands, which are the target organs of the HPA axis, were weighed on P24, P45 and P87 (Fig. 2A,B). The results showed a significant main effect of age (Left: F = 187.246, *P* < 0.001; Right: F = 234.038, *P* < 0.001); the bilateral relative adrenal weights were higher on P24 than on P45 and P87 for both groups (*P* < 0.001). However, no group effect (Left: F = 0.292, *P* = 0.591; Right: F = 2.505, *P* = 0.120) and interaction effect between age and group (Left: F = 0.866, *P* = 0.427; Right: F = 1.777, *P* = 0.180) were found in bilateral relative adrenal weights. In addition, the bilateral relative adrenal weights were significantly increased on P87 (Left: t = 4.47, *P < *0.001; Right: t = 5.696, *P* < 0.001) in the Needle group compared with the Tactile group.

#### Stress-triggered serum corticosterone and adrenocorticotropic hormone (ACTH) levels

As shown in Fig. 2C, the basal serum corticosterone level prior to neonatal treatment was 277.82 ± 50.28 ng/ml. There was no difference in basal serum corticosterone levels between the Needle and Tactile groups on P8 (t = 0.106, *P* = 0.917) and P15 (t = 1.202, *P* = 0.248). There was a decrease in the basal serum corticosterone level on P8 compared with P1 and P15. However, higher stress-triggered serum corticosterone levels persisted in both the Tactile and Needle groups after weaning (*P* < 0.05). The pattern of stress-triggered serum corticosterone levels changed with age (Fig. 2D: F(age) = 74.718, *P* < 0.001) and was different between the Tactile and Needle group (F(group) = 6.058, *P* = 0.016; F(age * group) = 11.735, *P* < 0.001). The stress-triggered serum corticosterone levels were more prominent on P24 than other time-points in the Tactile group (*P* < 0.01); this parameter was higher on P87 than other time points in the Needle group (*P* < 0.05). The stress-triggered serum corticosterone levels in the Needle group were significantly decreased on P24 (t = −4.066, *P* = 0.001) and increased at P87 (t = 2.561, *P* = 0.021) compared with the Tactile group.

The stress-triggered serum ACTH levels in the Needle group were decreased on P24 (Fig. 2E, t = −3.067, *P* = 0.007), which was consistent with the stress-triggered serum corticosterone levels on P24.

#### GR mRNA and protein expression in hippocampal tissue

**Figure 3:**
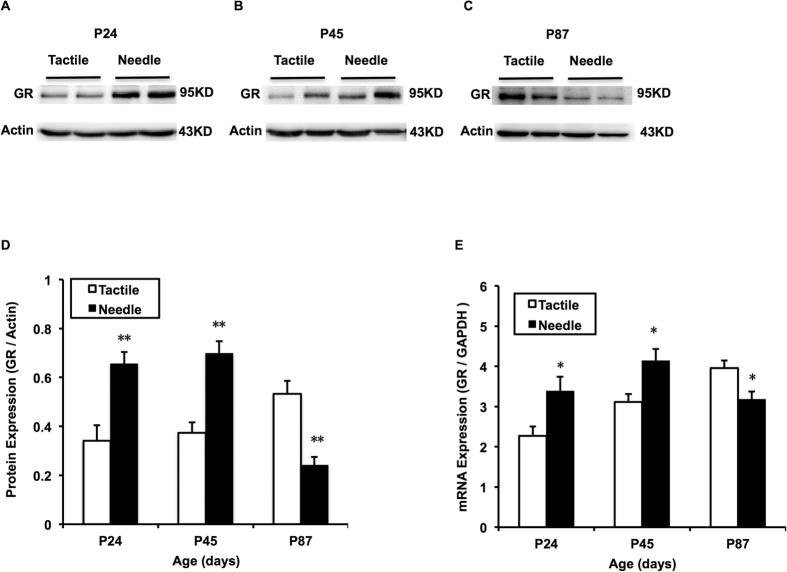
Hippocampal GR mRNA (**E**) and protein expression (**A**–**D**). The two-way ANOVA was used to detect group differences at different ages between the two groups. The LSD *post-hoc* test was used to detect group differences at each age. The GR mRNA levels are the ratio to the control group normalized by the corresponding GAPDH mRNA levels using the 2^−ΔΔct^ method. GR protein expression is expressed as fold change compared with the control. The bars represent the mean ± SEM. *P < 0.05 for Needle vs. Tactile (n = 10).

There was a significant difference between the groups in the expression of GR mRNA in hippocampal tissue (F = 4.304, *P* = 0.044) and in age (F = 6.029, *P* = 0.005). The hippocampal GR mRNA expression in the Tactile group increased over time (*P* < 0.05), whereas this tendency disappeared in the Needle group (Fig. 3E). Compared with the Tactile group, the hippocampal GR mRNA expression of the Needle group was significantly increased on P24 (t = 2.573, *P* = 0.019) and P45 (t = 2.891, *P* = 0.012), but decreased on P87 (t = −2.846, *P* = 0.013). The pattern of GR protein expression in hippocampal tissue was similar to the GR mRNA expression in both groups (F(group) = 8.147, *P* = 0.006; F(age) = 4.875, *P* = 0.011; F(group * age) = 25.395, *P* < 0.001), Fig. 3A–D).

#### GR protein immunohistochemistry (IHC) in hippocampal dorsal CA1

**Figure 4:**
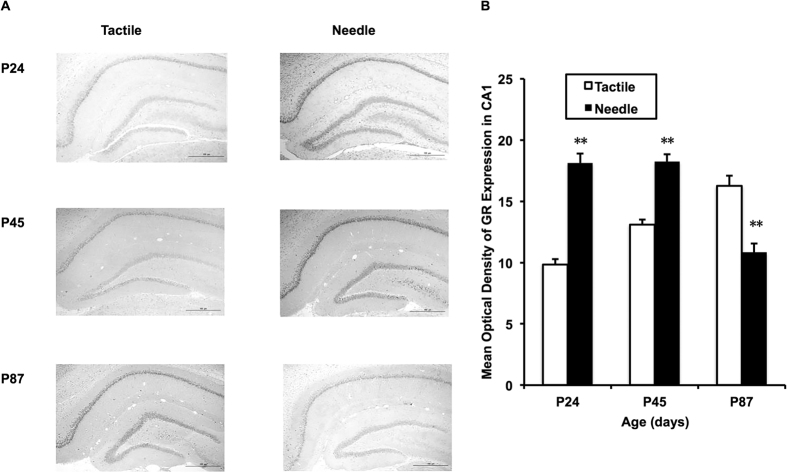
Hippocampal CA1 GR immunoreactivity. An image taken at objective magnification of 4x is shown (**A**). The two-way ANOVA test was used to detect group differences at different ages between the two groups (**B**). The LSD *post-hoc* test was used to detect group differences at each age. Data are expressed as the mean ± SEM. **P < 0.01 for Needle vs. Tactile (n = 6).

We chose to measure the mean optical density (MOD) of the hippocampal dorsal CA1 because multiple lines of evidence in rats have shown that the integrity of hippocampal CA1 field activity is necessary for the encoding and long-term storage of spatial information (Fig. 4A). As shown in Fig. 4B, there were significant differences in the MOD of GR protein immunoreactivity in the dorsal CA1 of the hippocampus among ages (F = 4.409, *P* = 0.022) and groups (F = 16.530, *P* < 0.001). Compared with the Tactile group, GR protein immunoreactivity in the hippocampal dorsal CA1 increased on P24 (t = 9.925, *P* < 0.001) and P45 (t = 6.389, *P* < 0.001) and decreased on P87 in the Needle group (t = −4.809, *P* < 0.001). Moreover, the increasing tendency of GR protein immunoreactivity over time in the Tactile group (*P* < 0.05) was disrupted in the Needle group.

## Discussion

In this study, a repeated needlestick protocol performed on rat pups was used to mimic the repeated heel/finger lances performed on preterm neonates during their stay in the NICU to investigate the effects of neonatal repetitive skin-breaking pain on later cognitive and emotional behaviors in both young and adult rats. Consistent with previous studies^8^,^39^, the neonatal repetitive needlestick protocol performed on rats resulted in persistent mechanical hypersensitivity. Moreover, we demonstrated that neonatal repetitive pain impaired spatial memory retention ability in prepubertal rats and dampened behavioral response to anxiety-provoking stimuli both in young and adult rats. Importantly, early painful experiences triggered a dynamic shift in HPA axis function, characterized by a dampened HPA axis reactivity with decreased stress responsiveness in adolescence and an increased HPA axis reactivity with elevated stress responses in adulthood. These results indicate that stress responsiveness in rats subjected to repeated neonatal pain has age-specific patterns, which are associated with altered hippocampal GR expression and HPA axis function.

First, we found that neonatal repetitive pain induced persistent mechanical hypersensitivity from childhood to adulthood. This inference corroborates other studies that have indicated that basal or secondary hyperalgesia occurs in young and adult animals exposed to early repetitive procedural pain^8^,^14^,^39^,^40^. In addition, this study indicates that neonatal repetitive pain might be associated with the alteration of pain pathways in later adulthood^8^. The direct contribution of HPA axis activity in modulating pain sensitivity is still a matter of debate in the scientific community and more studies are needed.

Second, we found that neonatal repetitive pain contributed to spatial-memory retention deterioration in prepubertal rats but not in adult rats. In the present study, prepubertal rats that had been previously subjected to repeated pain exposure exhibited longer latency and few crossings of the original platform location, whereas control rats maintained the same level of spatial memory during the probe trial. These results parallel clinical data reporting that poorer cognition in infants, toddlers and school-age children is associated with a higher number of skin-breaking procedures^15^,^16^,^41^. In animal models, however, the long-term cognitive effects of early pain are fraught with divisive findings. Bernardi *et al*. showed evidence of improved active avoidance performance in adult rats neonatally exposed to repeated painful stimuli (hot plate stimuli)^20^, whereas Schellinck *et al*. found no alteration of spatial learning ability in juvenile mice after repetitive needlestick exposure^21^. By contrast, Butkevich *et al*. confirmed the impairment of spatial learning in adolescent rats that had neonatally experienced repeated inflammatory pain^42^. The critical window when the pain occurs also plays a great role in the outcome. Therefore, the difference in spatial learning ability between the rats that experienced pain during P0-P7 (our study) and P8-P14 (Schellinck’s study^21^) indicates that pain experienced in preterm infants will have more adverse effects on cognitive development than that in term infants. However, there were no impairments on spatial learning and memory ability in adult rats that had experienced repetitive pain in our study. Further testing on emotional-related memory ability, such as fear conditioning, is needed.

Third, we assumed that the neonatal repetitive pain would result in increased anxiety-related behavior in both young and adult rats. In previous clinical studies, high numbers of skin-breaking procedures in the neonatal period predicted higher internalizing behaviors in toddlers and persisted to school age in children born very preterm^17^,^43^. The evidence from animal studies also suggested that the rats exposed to repetitive neonatal pain showed an increased preference for alcohol, increased anxiety and defensive withdrawal behavior, and prolonged social hypervigilance^14^. However, we found an increased distance travelled and more entries in the central area of the OF test in young rats, indicating a tendency of decreased anxiety-related behaviors or reduced sensitivity to aversive stimuli. Moreover, the tendency became more significant when the rats reached adulthood, manifested by more time and more distance in the inner area and less time spent in the outer area. The underlying mechanisms for anxiety in human and the anxiety-like behavior in rodents would be different. Rodents’ anxiety-like behavior in the OF test reflects the natural balance between the exploratory and escaping drives. Thus, entry into the central area could also be conceptualized as risk-taking behavior. In our experiment, rather than anxiety being reduced, the rats that experienced neonatal repetitive pain might not recognize the risk. Similarly, a previous study found that a single neonatal injury decreases behavioral sensitivity to aversive stimuli^19^. The risk-taking behavior and hyperactively could be interpreted as the classic features of mania in bipolar disorder, which is a severe psychiatric disorder. However, other emotional behaviors, such as depression-like behavior, still need further exploration in our animal model.

Finally, we confirmed our hypothesis that neonatal repetitive pain disrupted the development of the HPA axis from childhood to adulthood. There were lower levels of basal corticosterone for both groups during the SHRP after neonatal manipulation. The HPA axis development is particularly different in adolescence compared to childhood and adulthood^44^. According to previous studies, higher stress-triggered corticosterone levels occur on P30 because the negative feedback system of the HPA axis is immature until P30^44^,^45^,^46^. The shift in the stress-induced ACTH response appears to mature later, between 50 and 60 days of age^44^. In addition, the adrenal gland to body weight ratio significantly decreases in rats at each of the ages between 30 and 60 days of age^44^,^47^,^48^,^49^,^50^. However, there is a gradual increase in GR mRNA expression from birth onward, particularly in the case of DG and CA1 subfields^51^. Because the pain occurred during the SHRP in the present study, these age-related changes in the development of the HPA axis disappeared in rats that experienced early pain, manifested by a hyporesponse to stress in adolescence and a hyper-response to stress in adulthood. The hyporesponse of the HPA axis was demonstrated in prenatally stressed adolescent male rats^52^, but it was first reported in the neonatal pain model in adolescent male rats.

Previous findings have demonstrated an inverted-U-shaped relationship between circulating glucocorticoid levels and memory retrieval^53^,^54^,^55^, and moderate levels of corticosteroids are required for optimal cognitive function in humans and rodents^56^,^57^. More specifically, MRs are crucially involved in effective memory retrieval, whereas the activation of GRs may decrease retrieval performance^58^. The impaired spatial-memory retention ability in young rats might be associated with the elevated GR level in the hippocampus. The prepubertal shift toward stress hyporesponsiveness caused by an early pain experience will not only affect learning and memory but also alter synaptic structure and function in pathways mediating goal-directed behavior, thus influencing the behavioral and physiological responses to subsequent encounters. Further exploration, such as the sustained long-term potentiation (LTP) and long-term depression (LTD) in prepubertal rats, is required to assess the effects of early pain exposure on learning and memory function.

Overall, our results indicated that neonatal pain affected hippocampal neuronal plasticity, manifesting as increased GR expression, more efficiently transmitted negative glucocorticoid feedback to the HPA axis, down-regulated hypothalamic corticotropin-releasing hormone (CRH) and subsequent corticoid levels, and impaired learning and memory function in puberty. When the rats reached adulthood, the negative feedback of GR expression dampened, and corticoid levels increased, causing the risking-taking behavior. This age-specific pattern of biobehavioral changes indicates that the prepubertal stage is more vulnerable than adulthood, and the HPA axis “reset” regulated by the up- or down-regulation of GR expression in the hippocampus is dynamically programed by early life experiences^59^.

## Conclusion

In the present study, we demonstrated that neonatal repetitive needlestick exposure caused persistent mechanical hypersensitivity, spatial-memory retention impairment in prepuberty, and decreased response to anxiety-inducing stimuli both in prepubertal and adult rats. Moreover, young and adult rats that experienced neonatal repetitive procedural pain exhibited dysregulation of hippocampal GR activity and HPA axis function under stress. These findings indicate that repetitive pain during a critical period may cause severe consequences, with behavioral and neuroendocrine disturbances developing from prepuberty to adult life. For those who have experienced unmanaged neonatal pain, early evaluation and intervention to avoid emotional abnormality and learning difficulties are necessary.

## Materials and Methods

### Animals

This study was performed in strict accordance with the recommendations in the Guide for the Care and Use of Laboratory Animals of the National Institutes of Health. The protocol was approved by the Committee on the Ethics of Animal Experiments of Nanjing Medical University (Permit Number: NJMU/IACUC2011113001). All efforts were made to minimize animal suffering, to reduce the number of animals used and to utilize alternatives to *in vivo* techniques. Pregnant Sprague-Dawley rat dams (Vital River, Beijing, China) were purchased on day 14 of gestation and housed individually with food and water available *ad libitum*. The rats were maintained in the Experimental Animal Center affiliated with Nanjing Medical University, under controlled light (0600 h–1800 h) and temperature (22 ± 2 °C) conditions.

### Experimental design

Because preterm infants are subjected to either painful procedures or non-painful tactile stimulation in clinical situations and because our objective is to compare the long-term impacts of painful procedures and gentle tactile stimulation performed on the rat pups, a control group was not included. Repeated neonatal pain was induced similarly to the procedure described in a previous study^14^. Briefly, the stimulation paradigm for the pain group (Needle) was as follows: for each rat pup, one of its four paws received a needlestick every 6 hours, in the order of left hindpaw, right hindpaw, right forepaw and left forepaw. The needlestick was automatically performed by a blood glucose sampling device (Accu-chek^®^ softclix, Roche, Germany) at a depth setting of “4”, using a sterile 28-gauge lancet (Accu-chek^®^ softclix, Roche, Germany). Before each stimulation, a new sterile lancet was installed on the device to prevent cross-contamination and subsequent local inflammation. The needle was quickly inserted into the mid-plantar area, which is the thickest area of the paw, on a warm surface, in case the lancet penetrated the entire paw. The bleeding was stopped by the application of a cotton-tipped swab and lasted no more than a few seconds. Rat pups were returned to their dams between consecutive stimuli. Simultaneously, rat pups of the control group (Tactile) received a tactile stimulus. The tactile stimulation was applied gently to the mid-plantar area of the corresponding paw using a cotton-tipped swab, at same interval and same paw order as the Needle group. The separation time of the rat pups from their dams was no more than 5 minutes for either group to avoid the effects of maternal separation and neonatal handling^60^,^61^. The neonatal stimulation for both groups lasted for 8 days, from day of birth (P0) to postnatal day 7 (P7).

**Figure 5:**
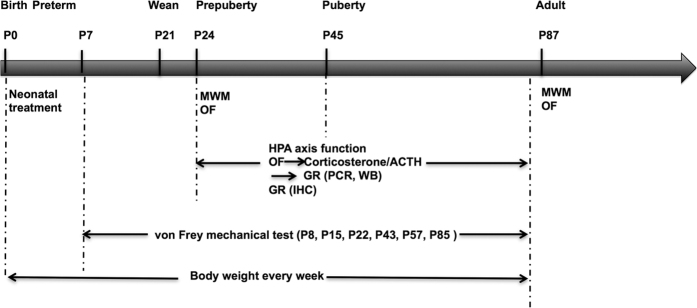
Experimental protocol. Within 24 h of birth (P0), male rat pups were randomly subjected to neonatal treatment, which lasted until P7. The Needle group received a needlestick performed on one of each rat pup’s four paws by turns at 6-hour intervals; at the same time, a cotton-tipped swab was applied to the corresponding paw of the rats in the Tactile group. All pups were then left undisturbed and weaned at P21. Mechanical sensitivity was tested on P8, P15, P22, P43, P57 and P85. On P24-P30 and P87-93, rats were submitted to either the Morris water maze (MWM) test or exploring in an open field (OF) for 30 min. They were then anesthetized for blood sampling and sacrificed for PCR and Western blotting (WB) or were transcardially perfused for immunohistochemistry (IHC). Body weight was measured every week.

**Figure 6:**
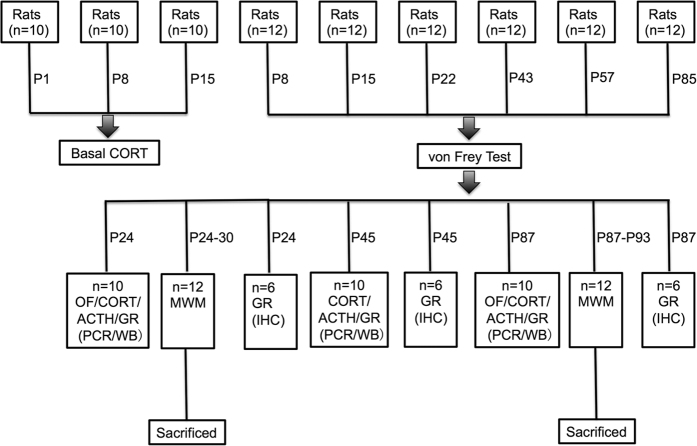
Number of rats used for each experiment. Corticosterone (CORT), open field test (OF), adrenocorticotropic hormone (ACTH), glucocorticoid receptor (GR), Western blotting (WB), Morris water maze test (MWM), immunohistochemistry (IHC).

In the present experiment, only male pups were included in our experiment. The litters were standardized to 10 pups per dam. Each dam fostered 5 Needle group rat pups and 5 Tactile group pups. The rat pups were randomly assigned to neonatal treatment and then randomly distributed to different dams. All litters were kept undisturbed after neonatal stimulation except for routine bedding changes. The pups were weaned on P21 and housed three per cage post-weaning until further testing. Body weight was measured using an electronic scale (sensitivity, 0.1 gram) every week until the rats were euthanized. As a whole, 29 litters were generated, and a total of 204 male rats was included in the present study. There were 102 rats in the Needle group and 102 rats in the Tactile group. Figure 5 shows the experimental protocol and Fig. 6 shows the number of rats used for each experiment.

### Behavioral tests

The behavioral tests were performed during the daytime (0900 h–1230 h) at room temperature (22 ± 2 °C); the animal order was randomized, and the observers were blinded to the neonatal treatment. Before the day of behavioral testing, the rats were allowed to acclimate to the test room for 24 hours. For von Frey mechanical tests at each time point, the rats from both groups were different from those used at other time points to avoid hypersensitivity. Similarly, the rats used for MWM and OF tests at each time points were also different from the other time points, but all had been previously tested once by von Frey tests.

### von Frey mechanical tests

On days P8, P15, P22, P43, P57 and P85, the nociceptive threshold to mechanical stimulation was determined using a von Frey Touch-Test^TM^ Sensory Evaluator (Stoelting Co, USA). A total of 12 rats from the Needle group and 12 rats from the Tactile group were tested using the sampling without replacement method at each time point. On the testing day, 6 rats were loaded individually into Plexiglas chambers resting on a 6 mm wire grid each time. After a 30-minute acclimation period, the mechanical withdrawal threshold was measured by applying a series of 20 calibrated von Frey filaments to the mid-plantar surface of the hindpaw. The threshold force of the paw was determined by the up-down method^62^. Positive nociceptive behaviors such as a brisk withdrawal of the paw, an attack or an escape reaction were elicited in 3 out of 5 trials (>50%). With an interval of 5 minutes between each application, sensitization could be effectively avoided. The other hindpaw was tested in the same manner. The MWT was defined as the lowest filament to evoke a > 50% withdrawal rate.

### MWM test

The MWM test was used to examine spatial learning and memory. A total of 12 Needle group rats and 12 Tactile group rats were tested for each time point. At P24-P30, the rats are equivalent to prepuberty, and the rats at P87–93 reach similar brain maturity as adults. A black water tank (150 cm in diameter) filled with water was positioned in the middle of a normally lit testing room with distant visual stimulFour points equally spaced along the circumference of the tank were arbitrarily assigned as northeast (NE), northwest (NW), southeast (SE) and southwest (SW). A black platform (10 cm in diameter) was submerged 2 cm beneath the water surface at the fixed location of SW. Water temperature was maintained at 20 ± 1 °C. The performance of the rats was automatically tracked using a video tracking system (Top View Animal Behavior Analyzing System; Clever Sys. Inc., USA).

The day before training, each rat was allowed to freely swim for 60 seconds without the platform for adaption. During the acquisition phase, the platform was kept invisible beneath the water, and all rats were subjected to four trials per day for five consecutive days. For each trial, the rat was placed in the water facing the wall of the pool at one of three starting points (NE, NW, SE) in a random order; the SW quadrant, where the platform was located, was always the last starting point used. All rats were allowed on the platform for 10 seconds. Rats that failed to locate the platform within 60 seconds were guided to it. Latency (actual swim time from a starting point to the platform), distance (actual swim distance from the starting point to the platform) and swim speed (path length divided by the latency) were calculated. Twenty-four hours after the acquisition phase, the rats were subjected to a probe trial to assess memory with the platform removed (60 seconds). The dependent measures were latency (first trial latency to find the platform), crossing index (number of platform site crossings), time spent in the target quadrant where the platform used to be (time spent in the SW quadrant), time spent in the opposite quadrant (time spent in the NE quadrant) and swim speed. At the end of each session of the test day, rats were towel dried and returned to their home cages.

### OF test

The OF test was used to evaluate anxiety-like behavior and locomotor activity. A total of 10 Needle group rats and 10 Tactile group rats were tested for each time point. The arena consisted of a blue square plastic floor (50 cm × 50 cm) surrounded by a blue 50 cm wall and was brightly lit white. The dimension of the central area and peripheral area within the OF was 125 cm^2^ and 125 cm^2^, respectively, thus the side length of the center area was 11.18 cm. The behavioral system constitutes four arenas (Clever Sys. Inc., USA); thus, each time 4 rats were simultaneously and gently placed in the center of each arena facing the same direction. The performance of the rats was tracked automatically using a video tracking system (Top View Animal Behavior Analyzing System; Clever Sys., Inc., USA). The arenas were thoroughly cleaned with 75% ethanol between each session and were not used until the odor volatilized. The following variables were recorded during the first 10-minute session: inner time (time spent in the central area), outer time (time spent in the peripheral area), total distance (actual locomotor path in the whole area), inner distance (%) (actual locomotor path in the central are divided by total distance), total crossing (number of entries into the peripheral and central area) and inner crossing (%) (number of entries into the central area divided by total crossing).

### HPA axis function

For male rats, the postnatal week 3 is designated as the weaning period, the postnatal weeks 6–7 are the puberty period, and the postnatal week 8 is the post-puberty period^46^. Therefore, P24, P45 and P87 represent the prepuberty, puberty and adulthood periods of rats, and the examination of HPA function was performed at the three stages.

### Stress-triggered hormones

After 30 minutes of exploration in an open field to trigger stress hormones, rats were immediately anesthetized with chloral hydrate (300 mg/kg body weight, IP) at 0900 h–1000 h following an overnight fast (12 hours), and blood samples were obtained from the right ventricle and collected in the absence of EDTA or heparin. After the blood samples stood for 1 h at room temperature (22 ± 2 °C), the serum started to precipitate. Then, the blood samples were centrifuged (2000 × *g*, 20 °C, 15 minutes), and the separated serum was stored at −70 °C for subsequent examination. In addition, on P1, P8 and P15, basal serum corticosterone levels were obtained at 0900 h–1000 h following a 2-h fast without any stimulation.

Serum corticosterone was measured using an enzyme-linked immunosorbent assay (ELISA) kit (ab108821, Abcam, Cambridge, UK), for which the sensitivity limit was 0.3 ng/ml, the intra-assay coefficient of variation was 5.0%, and the inter-assay coefficient was 7.2%. Serum ACTH was analyzed by electrochemiluminescence immunoassay (ECLIA) using a Roche Cobas^®^ 6000 Analyzer (Roche Diagnostics GmbH, Mannheim, Germany). A total of 10 Needle group rats and 10 Tactile group rats were tested for each time point.

### Relative weight of the adrenal glands

The bilateral adrenal glands were carefully dissected and weighed on an electronic scale (sensitivity, 0.0001 grams). Adrenal weights were expressed as milligrams per gram of body weight to correct the weight of the gland in relation to the weight of rat. A total of 10 Needle group rats and 10 Tactile group rats were tested for each time point.

### GR mRNA expression

The heads were quickly removed on ice, and the bilateral hippocampi were separated, snap-frozen in liquid nitrogen and stored at −80 °C until gene expression and Western blot analysis. RNA was counterbalanced for bilateral hippocampal tissues and isolated with TRIzol (Invitrogen, Carlsbad, CA, USA) according to the manufacturer’s instructions. RNA quality was evaluated using a NanoDrop 1000 Spectrophotometer (Thermo, USA). The integrity of total RNA was assessed using agarose gel electrophoresis, and cDNA was synthesized using a Transcriptor First Strand cDNA Synthesis Kit (Roche Diagnostics GmbH, Germany) with 1.0 μg of the RNA sample, as described by the manufacturer. PCR amplification using glyceraldehyde-3-phosphate dehydrogenase (GAPDH) primers on a subset of the cDNA samples confirmed successful reverse transcription. Real-time PCR was performed using the SYBR GREEN (Roche Diagnostics GmbH, Germany) Real-time PCR system (ABI-7500, Singapore) with the following program: 50 °C for 2 minutes, 95 °C for 10 minutes and 40 cycles of 95 °C for 15 seconds, and 60 °C for 1 minute. The mRNA levels were normalized to the corresponding GAPDH mRNA levels. Comparative gene expression to the Tactile group was analyzed with a 2^−ΔΔct^ method. The primer sequences were as follows (Invitrogen™, Life Technologies, USA): GR, forward 5′-GGG TAC TCA AGC CCT GGA ATG-3′, reverse 5′-CCC GTA ATG ACA TCC TGA AGC T-3′; GAPDH, forward 5′-GGC TCT CTG CTC CCT GTT CTA-3′, reverse 5′-CGT CCG ATA CGG CCA AAT CCG T-3′. A total of 10 Needle group rats and 10 Tactile group rats were tested for each time point.

### GR protein Western blotting

GR protein was determined by Western blotting. Total protein was counterbalanced for bilateral hippocampal tissues and was homogenized in ice-cold radio immunoprecipitation assay (RIPA) buffer with phenylmethanesulfonyl fluoride (PMSF) to make whole cell lysates. Protein concentrations were determined using a Pierce BCA protein assay kit with bovine serum albumin as the standard (Thermo Fisher Scientific, Rockford, IL, USA). A total of 40 μg of protein was loaded into each well of a 10% SDS–PAGE gel, separated, and transferred onto nitrocellulose (NC) filter membranes (0.45 mm; Millipore, Billerica, USA) at 4 °C. The membranes were blocked in 5% non-fat milk for 1.5 hours at room temperature and then blotted with a primary antibody against GR (rabbit polyclonal, Santa Cruz Biotechnology, Santa Cruz, CA, USA, sc-1004, 1:250)^63^ or actin (rabbit polyclonal, Santa Cruz Biotechnology, Santa Cruz, CA, USA, sc-7210, 1:1000)^64^ overnight at 4 °C. After washing four times (10 minutes/wash) in phosphate-buffered saline containing Tween-20 (PBST) at room temperature, the membranes were incubated with the secondary antibody (anti-rabbit IgG, Santa Cruz Biotechnology, Santa Cruz, CA, USA) at a 1:1000 dilution for 1 hour. After being washed four times (10 minutes/wash) in PBST at room temperature, immunoreactive bands were visualized using a ChemiDoc XRS + Imaging System (Bio-Rad Molecular Image, USA) with electrochemiluminescence (ECL) detection reagent (Amersham, Buckinghamshire, UK). Densitometric analysis was performed using Quantity One software (Bio-Rad). A total of 10 Needle group rats and 10 Tactile group rats were tested for each time point.

### GR protein IHC

Rats were deeply anesthetized with chloral hydrate (300 mg/kg body weight, IP) at 0900 h–1000 h and perfused transcardially with 37 °C 0.9% saline, followed by 4 °C 4% paraformaldehyde in 0.01 M sodium phosphate buffer (PBS, pH 7.4). Brains were removed, post-fixed overnight in 4% paraformaldehyde and embedded in paraffin. For immunostaining, the paraffin blocks were coronally cut into 3 μm sections of hippocampi (Cryostat 2800 Frigocut-E, Leica Instruments), mounted on glass slides (Superfrost^®^ Excell™, Thermo, HA, USA) and stored at −20 °C until usage. Glass slides were thawed for approximately 2 hours at room temperature. The sections were washed in phosphate-buffered saline (PBS, 3 × 3 minutes) and then treated with blocking buffer (containing 2% goat serum and 3% hydrogen peroxide) for 1 hour at 37 °C. After washing with PBS (3 × 3 minutes), the sections were incubated with anti-GR antibody (rabbit polyclonal, Santa Cruz Biotechnology, Santa Cruz, CA, USA, sc-1004) at a dilution of 1:200 at 4 °C overnight. Following incubation with the primary antibody, the sections were washed with PBS (3 × 3 minutes) and incubated with the secondary antibody (MaxVision^TM^ HRP-Polymer anti-Rabbit IHC Kit, MXB, China) at room temperature for 15 minutes. After washing with PBS (3 × 3 minutes), the immunoreactivity of each section was visualized by treatment with hydrogen peroxide and 3,3′-diaminobenzidine tetrahydrochloride (DAB) (Invitrogen™, Life Technologies, USA) and observed with a microscope (Olympus, BX50, Japan). All images were captured by digital sight camera (Nikon, Japan) with an objective magnification of 4x. The MOD of GR in the dorsal hippocampal CA1 was measured using the Image-Pro^®^ Plus image analysis system (Media Cybernetics, Silver Spring, MD, USA). We followed the instruction of the analysis system with regard to adjusting the immunoreactivity of the hippocampus to non-specific background staining. The total optical density of the dorsal hippocampal CA1 divided by the area of dorsal CA1 is the MOD. The average MOD was calculated by bilateral hippocampi in three sections per rat and 6 rats per group.

### Statistics

Analysis was performed in SPSS version 16.0 for Windows (SPSS, Inc., Chicago, IL, USA). Values are expressed as the mean ± SEM. Repeated-measures univariate analysis of variance (ANOVA) was conducted to determine the significance of differences in body weight and parameters of the MWM during the acquisition phase between the Needle and Tactile groups. Two-way ANOVA was performed to compare the effects of group and training on latency, as well as the effects of group and quadrant on time spent in the MWM probe phase, mechanical withdrawal threshold and HPA axis function. Group differences at certain time points were determined by the least significant difference (LSD) *post-hoc* test. The independent t-test was used to detect group differences in crossing index and speed in the MWM probe phase, as well as in the OF test. The paired t-test was used to examine the difference in latency between the D5 and the probe phase, as well as the difference in the time spent between the target and opposite quadrants in the MWM for each group. *P* values less than 0.05 (two-tailed) were considered significant.

## Additional Information

**How to cite this article**: Chen, M. *et al*. Neonatal repetitive pain in rats leads to impaired spatial learning and dysregulated hypothalamic-pituitary-adrenal axis function in later life. *Sci. Rep.* **6**, 39159; doi: 10.1038/srep39159 (2016).

**Publisher’s note:** Springer Nature remains neutral with regard to jurisdictional claims in published maps and institutional affiliations.

## Figures and Tables

**Table 1:** Body weight during the studying period.

Age	Tactile	Needle	*P*
P0	6.52 ± 0.08	6.44 ± 0.07	0.508
P8	17.14 ± 0.25	14.82 ± 0.24	< 0.001**
P15	32.92 ± 0.43	30.12 ± 0.54	< 0.001**
P22	54.22 ± 0.68	52.47 ± 0.80	0.103
P29	97.68 ± 1.74	93.61 ± 1.60	0.088
P36	143.55 ± 1.36	140.78 ± 1.74	0.208
P43	198.96 ± 2.94	204.90 ± 2.80	0.148
P50	245.54 ± 3.27	244.54 ± 3.28	0.831
P57	289.60 ± 3.93	279.52 ± 4.03	0.081
P64	324.29 ± 7.29	318.13 ± 5.99	0.515
P71	356.30 ± 7.23	354.75 ± 5.66	0.865
P78	378.76 ± 8.30	377.19 ± 6.28	0.879
P85	402.02 ± 9.16	395.04 ± 6.13	0.514

A repeated-measures univariate analysis of variance (ANOVA) model was used to compare the differences in body weight between the two groups from P0 to P85. The least significant difference (LSD) post-hoc test was used to evaluate the differences between the two groups at each age. Data are expressed as the mean ± SEM. **P < 0.01 for Needle vs. Tactile (n = 20).

**Table 2:** Mechanical withdrawal thresholds for the bilateral hindpaws of rats.

Age/groups	Left hindpaw		Right hindpaw	
	Tactile	Needle	Tactile	Needle
P8	1.72 ± 0.10	1.09 ± 0.13**	1.62 ± 0.10	1.17 ± 0.08**
P15	5.00 ± 0.33	3.95 ± 0.28**	4.80 ± 0.33	3.07 ± 0.42**
P22	4.97 ± 0.45	2.15 ± 0.40**	5.22 ± 0.43	1.78 ± 0.39**
P43	8.59 ± 0.78	4.19 ± 0.57**	6.85 ± 0.78	4.13 ± 0.48**
P57	18.83 ± 3.29	9.50 ± 0.88**	17.83 ± 2.70	9.25 ± 0.69**
P85	25.75 ± 4.90	9.94 ± 1.50**	25.92 ± 5.02	13.06 ± 1.84**

Two-way ANOVA was performed to determine the significance of differences in the mechanical withdrawal thresholds of the bilateral hindpaw between the two groups. The LSD post-hoc test was used to detect group differences at each age. Data are expressed as the mean ± SEM. *P < 0.05, **P < 0.01 for Needle vs. Tactile (n = 12).

**Table 3:** Open-field test.

Age	P24		P87	
Behavior/groups	Tactile	Needle	Tactile	Needle
Inner time (s)	10.55 ± 3.36	20.35 ± 3.48^#^	9.46 ± 1.84	15.61 ± 2.10^*^
Outer time (s)	587.70 ± 3.38	578.60 ± 3.39	590.18 ± 1.84	583.23 ± 2.06^*^
Inner distance (%)	5.64 ± 0.99	8.53 ± 0.83^*^	2.68 ± 0.54	5.06 ± 0.64^**^
Total distance (mm)	11299.44 ± 915.05	12975.77 ± 702.44	13465.64 ± 712.51	15371.12 ± 724.98
Inner crossing (%)	34.42 ± 3.44	45.02 ± 1.38**	41.64 ± 2.65	46.3 ± 1.14
Total crossing (n)	15.20 ± 2.43	19.35 ± 1.95	11.06 ± 1.83	19.78 ± 2.59*

Data are expressed as mean ± SEM. Student-t test was used to compare the group difference, *P < 0.05, **P < 0.01, ^#^*P* = 0.05 for Needle vs. Tactile (n = 10), n = number of occurrences.
